# From science to sleep: the promise of dual orexin receptor antagonists

**DOI:** 10.1055/s-0046-1827822

**Published:** 2026-10-06

**Authors:** Dalva Poyares, Ronaldo D. Piovezan, Sergio Tufik

**Affiliations:** 1 Universidade Federal de São Paulo, Departamento de Psicobiologia, Divisão de Medicina e Biologia do Sono, São Paulo, SP, Brazil.; 2 Adelaide University, College of Health, Adelaide, Australia.

The management of chronic insomnia has long been hampered by the limitations of traditional hypnotics, the risks of physiological dependence, rebound insomnia, accidents, falls and fractures, and cognitive impairment. The emergence of dual orexin receptor antagonists (DORAs) represents a paradigm shift, moving the therapeutic focus from non-specific sedation to the targeted regulation of the orexin system. The systematic review and meta-analysis by Araujo LMM et al, 1 focusing on the long-term DORA use, provides evidence that this class of drugs potentially offers sustained efficacy for up to 12 months, challenging the historical reliance on short-term pharmacological interventions and the lack of safety in the long term pharmacological treatment of chronic insomnia.

The findings confirm that suvorexant, lemborexant, and daridorexant provide consistent improvements in subjective sleep parameters, including total sleep time and sleep onset latency, without the major safety concerns associated with benzodiazepines or Z-drugs. However, the data also suggest that these agents may not be interchangeable. While all three demonstrate efficacy, they exhibit distinct dose–response profiles. Suvorexant at higher doses (40/30 mg) shows robust efficacy but is associated with a higher incidence of adverse events at the six-month mark and dose-dependent sedation. 2 Discontinuation rates were higher for the highest doses of Lemborexant (10 mg) and Suvorexant (40/30 mg). 1

Lemborexant demonstrates dose-dependent improvements in sleep efficiency, yet clinicians must weigh these benefits against an increase in fatigue and somnolence. Similarly, daridorexant's efficacy is most pronounced at the 50 mg dose, which simultaneously correlates with a higher risk of adverse events, although mild. This suggests that while DORAs are a safer alternative to traditional hypnotics, they require careful, patient-specific titration to balance therapeutic gain against tolerability.

Within the current arsenal of insomnia treatments, DORAs occupy a new space. By blocking the wake-promoting signals of the orexin system rather than inducing generalized CNS depression, they offer a more physiological approach to sleep maintenance and hyperarousal. 3 This mechanism is particularly promising for long-term use, as the meta-analysis indicates a safety profile comparable to placebo regarding serious adverse events, despite the persistent reporting of somnolence.

However, a significant gap remains regarding the use of DORAs in comorbid insomnia. The current body of randomized controlled trials largely excludes patients with psychiatric comorbidities or complex sleep disorders. This is a critical limitation applied to all other drugs approved for insomnia. Given that insomnia is frequently a symptom of underlying psychiatric conditions, such as depression or anxiety, the safety and efficacy of DORAs in these populations remain an open question. Could their non-sedative mechanism make them the ideal hypnotic for patients with psychiatric vulnerabilities, or do they carry unforeseen risks in these groups?

Nonetheless, DORAs have also emerged as an alternative to treat insomnia in special populations, such as those with dementia, with a probable higher safety profile in comparison to traditional hypnotics. Some studies in patients with insomnia and dementia, probable Alzheimer disease, using polysomnography and actigraphic data, show that suvorexant can improve total sleep time and wake after sleep onset in mild-to-moderate AD dementia without worsening underlying cognition 4 5 . Furthermore, lemborexant can consolidate nighttime sleep and reduce nocturnal activity in patients with irregular sleep-wake rhythm disorder associated with AD. 6

Moving forward, the field requires larger, long-term and pragmatic trials that include heterogeneous populations, specifically those with psychiatric and medical comorbidities, who also are more susceptible to drug interactions due to the prolonged use of multiple medications for chronic conditions. While the current evidence establishes DORAs as a reliable, long-term option for primary insomnia, their true potential in the broader landscape of sleep medicine will only be achieved when we understand how they perform in the complex, real-world patients who are most in need of safe, sustainable sleep therapy. 7

## References

[1] Araujo L MM Sousa A D Vieira C C Long-term use of dual orexin receptor antagonists for the treatment of insomnia: a systematic review and meta-analysis Arq Neuropsiquiatr 2026 84 07 s00461827049 10.1055/s-0046-1827049 PMC13472714 42595319

[2] Citrome L Suvorexant for insomnia: a systematic review of the efficacy and safety profile for this newly approved hypnotic - what is the number needed to treat, number needed to harm and likelihood to be helped or harmed? Int J Clin Pract 2014 68 12 1429 1441 10.1111/ijcp.12568 25231363

[3] Żełabowski K Petrov W Wojtysiak K Targeting the Orexin System in the Pharmacological Management of Insomnia and Other Diseases: Suvorexant, Lemborexant, Daridorexant, and Novel Experimental Agents Int J Mol Sci 2025 26 17 8700 10.3390/ijms26178700 PMC12429101 40943625

[4] Herring W J Ceesay P Snyder E Polysomnographic assessment of suvorexant in patients with probable Alzheimer's disease dementia and insomnia: a randomized trial Alzheimers Dement 2020 16 03 541 551 10.1002/alz.12035 PMC7984350 31944580

[5] Ahmed Abbasi A Norouziyan F Moradikor N Role of suvorexant in Alzheimer's disease: Targeting sleep, orexin signaling, and disease pathophysiology J Alzheimers Dis 2026 1387287726146116113872877261461161:10.1177/13872877261461161 42312432

[6] Moline M Bsharat M Cheng J Long-term safety and efficacy of lemborexant in subjects with irregular sleep-wake rhythm disorder and Alzheimer's disease dementia Alzheimer's Dement. 2020 16 e039039 10.1002/alz.039039 33336219

[7] Heidenreich S Ross M Chua G N Seboek Kinter D Phillips-Beyer A Preferences of patients for benefits and risks of insomnia medications using data elicited during two phase III clinical trials Sleep 2022 45 11 zsac204 10.1093/sleep/zsac204 PMC9644122 36054921

